# CYB5R1 links epithelial-mesenchymal transition and poor prognosis in colorectal cancer

**DOI:** 10.18632/oncotarget.8912

**Published:** 2016-04-22

**Authors:** Christine Woischke, Cristina Blaj, Eva Marina Schmidt, Sebastian Lamprecht, Jutta Engel, Heiko Hermeking, Thomas Kirchner, David Horst

**Affiliations:** ^1^ Pathologisches Institut, Ludwig-Maximilians-Universität München, München, Germany; ^2^ Tumorregister München, Institut für medizinische Informationsverarbeitung, Biometrie und Epidemiologie, Ludwig-Maximilians-Universität München, München, Germany; ^3^ German Cancer Consortium (DKTK), Heidelberg, Germany; ^4^ German Cancer Research Center (DKFZ), Heidelberg, Germany

**Keywords:** CYB5R1, colorectal cancer, EMT, survival, drug metabolism

## Abstract

Colorectal cancers show significant tumor cell heterogeneity within the same core genetic background. Epithelial-mesenchymal transition (EMT) is an important functional aspect of this heterogeneity and hallmark of colorectal cancer progression. Here, we identify CYB5R1, an enzyme involved in oxidative stress protection and drug metabolism, as an indicator of EMT in colon cancer. We demonstrate high CYB5R1 expression in colorectal cancer cells undergoing EMT at the infiltrative tumor edge and reveal an extraordinarily strong association of CYB5R1 expression with two core EMT gene expression signatures in a large independent colon cancer data set from The Cancer Genome Atlas (TCGA). Furthermore, we demonstrate that CYB5R1 is required for an infiltrative tumor cell phenotype, and robustly linked with poor prognosis in colorectal cancer. Our findings have important implications for colon cancer cells undergoing EMT and may be exploited for diagnostic and therapeutic purposes.

## INTRODUCTION

Colorectal cancer (CRC) is a common malignancy with worldwide incidence rates ranking second in men and third in women among all diagnosed cancers [1]. The genetic basis of CRC is quite well understood with a spectrum of accumulating mutations in key signaling pathways such as WNT, MAPK and TP53 that transform normal colonic mucosa into invasively growing tumors [2]. Although key mutations are mostly clonal within each tumor and affect most tumor cells [3], CRCs are composed of heterogeneous tumor cell subpopulations with distinct phenotypes and functions [4]. Tumor cell heterogeneity can be observed in most CRCs on the histological level, where cancer cells at the leading tumor edge appear undifferentiated and infiltrate surrounding stromal tissue [5]. In contrast, cancer cells of the tumor center are more differentiated and typically form glandular secondary structures [6]. This illustrates that tumor cell heterogeneity in part is reflected in differential tumor cell morphology within the same genetic background.

Specifically infiltrative tumor cells at the leading tumor edge are suspected drivers of malignant progression in CRC [4]. In addition to an undifferentiated morphology, this tumor cell subset has been demonstrated to partially lose epithelial characteristics and gain mesenchymal traits, a process termed epithelial-mesenchymal transition (EMT) [7]. Known inducers of EMT in CRC are transcription factors such as ZEB1 and SNAIL1 which promote colon cancer cell invasion while increasing the expression of mesenchymal markers including Vimentin, Fibronectin and Laminin gamma 2 [8–10]. At the same time, the expression of the epithelial cell adhesion molecule E-Cadherin is reduced, apparently allowing tumor cells to detach from the tumor mass during stromal infiltration [11]. By promoting invasion, EMT contributes to tumor progression and metastasis, and is associated with poor prognosis in colorectal and other cancers [12, 13]. Moreover, EMT has been linked to putative cancer stem cell characteristics and appears to promote chemo- and radiotherapy resistance of colon cancer cells [14].

Since colon cancer cells undergoing EMT are crucial for CRC progression, their detection and characterization might aid the development of therapies that specifically target EMT, reduce therapy resistance, and improve patient prognosis [15]. Here, we identify high expression of NADH-cytochrome b5 reductase 1 (CYB5R1), an enzyme about which relatively little is known, and which is part of an enzyme family involved in oxidative stress reactions and drug metabolism [16], in CRC cells undergoing EMT. Moreover, CYB5R1 expression directly links EMT and poor prognosis in this tumor entity. Our findings not only indicate high biomarker potential of CYB5R1, but importantly identify a potential drug target in tumor cells undergoing EMT at the infiltrative tumor edge of colorectal cancer.

## RESULTS

### CYB5R1 marks colorectal cancer cells undergoing EMT at the infiltrative tumor edge

**Figure 1:**
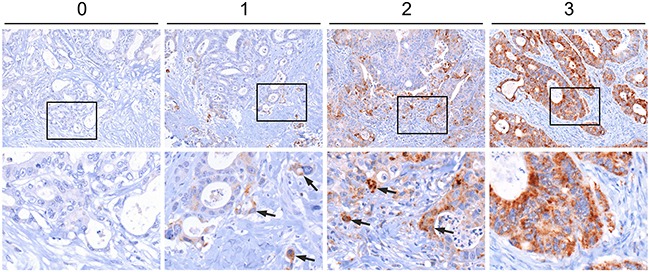
Immunostaining of CYB5R1 in colorectal cancer Assessment of CYB5R1 staining in a collection of 221 primary colorectal cancers. Tumors were assigned scores from 0 (no CYB5R1 staining) to 3 (strong CYB5R1 staining in most tumor cells). *Arrows* indicate CYB5R1 staining in tumor cells at the infiltrative edge. Images in *lower panels* show higher magnifications of the areas boxed in *upper panels*.

**Figure 2:**
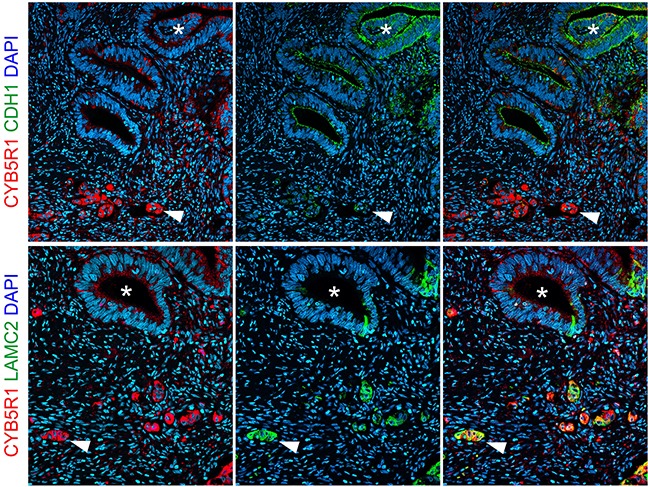
Confocal immune fluorescence for CYB5R1 Staining of individual colorectal cancer cases indicates loss of E-Cadherin (CDH1) expression in tumor cells with strong CYB5R1 expression (*upper panels*), and co-localization of CYB5R1 with Laminin gamma 2 (LAMC2, *lower panels*). *Arrowheads* indicate infiltrative colon cancer cells at the leading tumor edge. *Asterisks* indicate tumor cells with glandular differentiation. *Right panels* show composites of *left* and *mid panels*.

To learn about the significance of CYB5R1 in CRC, we initially characterized its expression in normal mucosa and in a collection of 221 CRC cases by immunohistochemistry. Normal colonic mucosa, adjacent to cancer tissues, demonstrated faint CYB5R1 expression that was confined to few epithelial cells at the crypt base (data not shown). In CRC, staining revealed a spectrum of CYB5R1 expression, ranging from negative in 31 cases (14%, score 0), through weak and moderate expression in 150 cases (68%, score 1) and 36 cases (16%, score 2), respectively, to strong expression in 4 cases (2%, score 3, Figure 1). Interestingly, while CYB5R1 marked most tumor cells in cases with strong expression by definition, cancers with weak or moderate CYB5R1 expression showed a distinct intratumoral distribution of this protein: In most of these tumors (83%), expression was pronounced at the infiltrative tumor edge, and was most strong in tumor cells that invaded the surrounding stroma by apparently detaching from the gland forming tumor mass (Figure 1). Surrounding stromal cells were negative or only weakly positive for CYB5R1. Since infiltrative colon cancer cells at the leading tumor edge are known to partially lose their epithelial phenotype [11], we next analyzed CYB5R1 expression and the expression of the epithelial cell adhesion protein E-Cadherin in individual cases by confocal immune fluorescence. Indeed, CYB5R1 labelled colon cancer cells with decreased or absent E-Cadherin expression, while gland forming tumor cells of the tumor center showed the opposite phenotype (Figure 2). Moreover, CYB5R1 co-localized with expression of the EMT marker Laminin gamma 2 (Figure 2). These findings suggested that CYB5R1 most strongly marked a subset of infiltrative, morphologically undifferentiated tumor cells undergoing EMT at the leading tumor edge of CRC.

### *CYB5R1* gene expression is strongly linked to EMT in colon cancer

**Figure 3:**
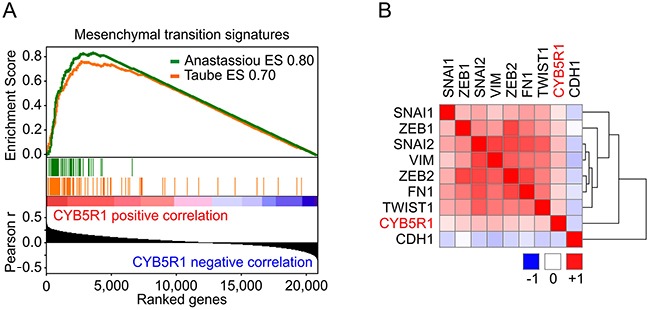
*CYB5R1* is linked to EMT in gene expression data of colon cancers from the TCGA **A.** Gene Set Enrichment Analyses for genes ranked by Pearson correlation (Pearson r) of expression to *CYB5R1* indicates enrichment for two core EMT gene signatures [17, 18]. ES=enrichment score. p < 0.001. **B.** Heat map indicates clustering and positive correlation of *CYB5R1* expression with colon cancer relevant EMT markers and negative correlation with *CDH1*. Colors indicate Pearson r from −1 (*blue*) to 1 (*red*).

To further examine a possible link of CYB5R1 and EMT in colon cancer, we analyzed independent gene expression data of 457 colon cancer cases from The Cancer Genome Atlas (TCGA). In these data, Gene Set Enrichment Analyses (GSEA) revealed highly significant (p<0.001) correlations of *CYB5R1* mRNA expression and the expression of two published core EMT gene signatures [17, 18], strongly linking *CYB5R1* and EMT (Figure 3A). Moreover, markers that induce or indicate EMT in colon cancer were significantly overexpressed in tumors with high *CYB5R1* levels, including *ZEB1* (r=0.20, p<0.0001), *TWIST1* (r=0.22, p<0.0001), and *VIM* (r=0.29, p<0.0001) (Figure 3B). In contrast, the epithelial differentiation marker *CDH1* negatively correlated with *CYB5R1* in this data set (r=−0.15, p=0.001, Figure 3B). These findings further supported the idea that *CYB5R1* is an indicator of EMT and confirmed our *in situ* findings for CYB5R1 and E-Cadherin on the mRNA level in a large independent data set.

### CYB5R1 depletion reduces migration and invasion of colon cancer cells

**Figure 4:**
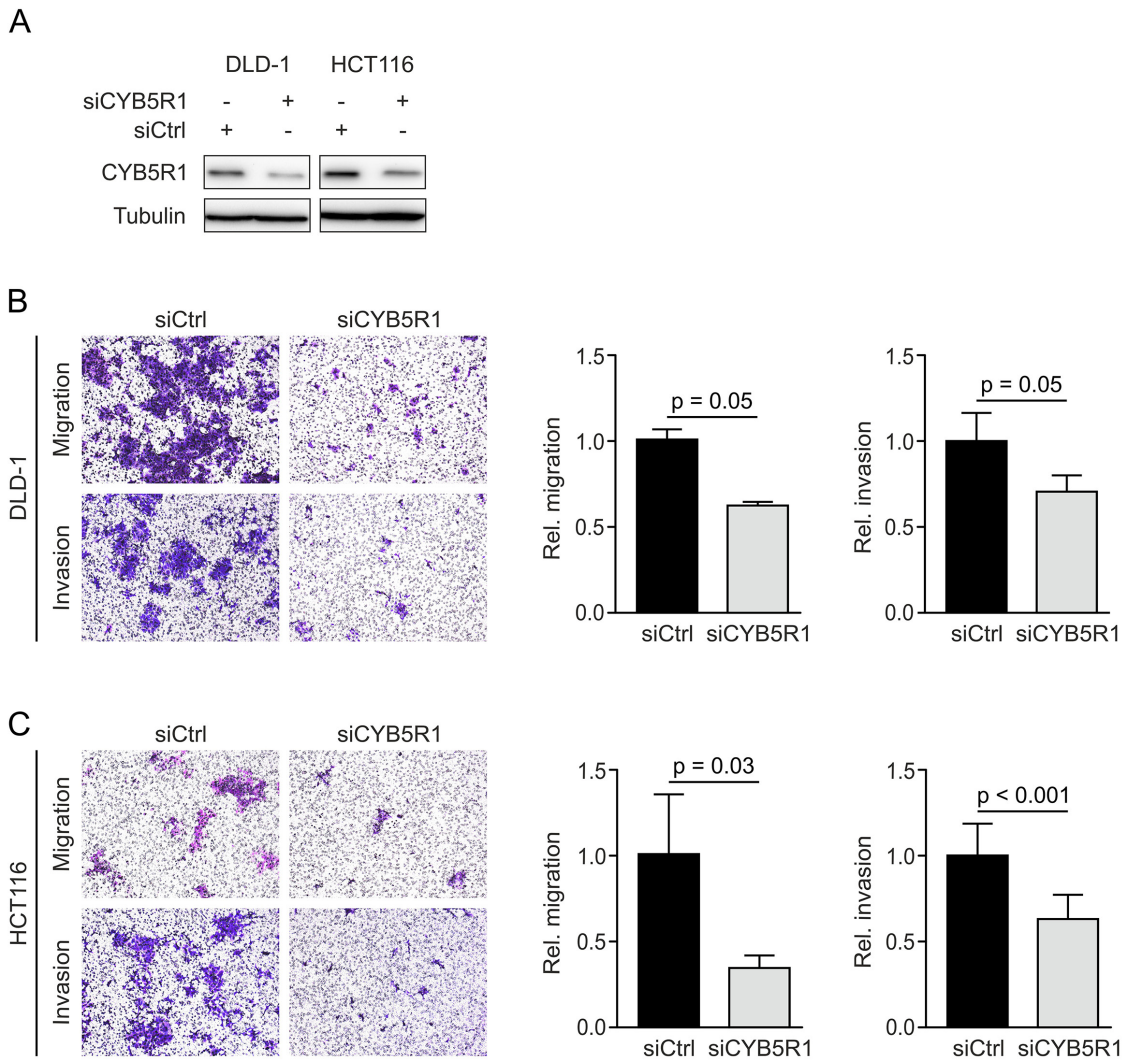
CYB5R1 depletion decreases migration and invasion of colon cancer cells **A.** Immunoblotting for indicated proteins on whole cell lysates of DLD-1 and HCT116 colon cancer cells harvested 48 h after transfection with *CYB5R1* or control (Ctrl) siRNA. **B–C.** Representative micrographs (*left panels*) and quantification (*right panels*) of migrated or invaded DLD-1 (B) and HCT116 (C) colon cancer cells in transwell assays. Data are mean ± SD, n ≥ 3, p-values are t test results.

Since EMT is linked to migratory and invasive tumor cell phenotypes in colorectal cancer, we next assessed the effects of CYB5R1 depletion on these malignant traits of colon cancer cells. We treated DLD-1 and HCT116 colon cancer cell lines with siRNAs specifically directed against *CYB5R1* mRNA, which resulted in reduction of CYB5R1 protein levels in both cell lines, when compared to control siRNA treatment (Figure 4A). We then seeded cells with and without CYB5R1 depletion in Boyden Chamber assays, and observed considerable decreases in transwell migration and invasion of both cell lines, while these effects were more pronounced in HCT116 than in DLD-1 colon cancer cells (Figure 4B, 4C). These findings suggested that CYB5R1 not only indicated EMT in colon cancer but also was functionally required for an EMT associated invasive and migratory colon cancer cell phenotype.

### CYB5R1 expression predicts poor prognosis of colorectal cancer patients

**Figure 5:**
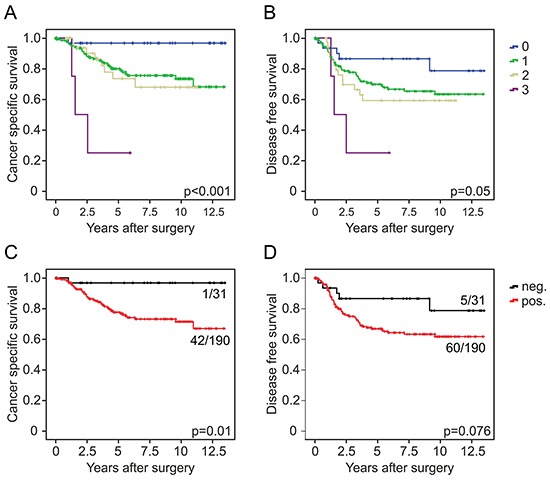
CYB5R1 indicates poor survival in colorectal cancer Kaplan-Meier plots for different CYB5R1 expression scores for tumor specific survival **A.** and disease free survival **B.** indicate significant poorer outcome with increasing CYB5R1 expression. Results for dichotomal CYB5R1 expression (negative and positive) are shown in **C.** and **D.** Significance p-values indicate log-rank test results. Ratios on curves indicate the number of events over the number of patients per group.

**Figure 6:**
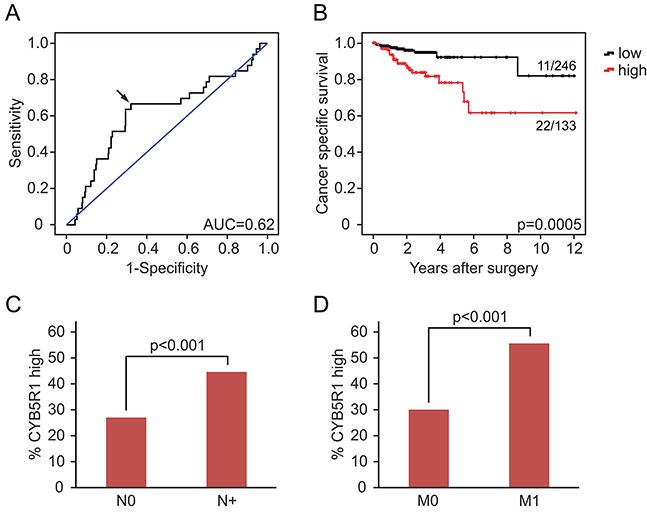
*CYB5R1* mRNA expression and survival in colon cancer data from the TCGA **A.** ROC curve for determining best discrimination thresholds of *CYB5R1* mRNA reads for survival prediction. Arrow indicates chosen value for binary classification. AUC=Area under curve. **B.** Kaplan-Meier statistics for binary (low and high) classified *CYB5R1* expression indicate shorter tumor specific survival for *CYB5R1* high. Significance p-values indicate log-rank test results. Ratios on curves indicate the number of events over the number of patients per group. **C, D.** Cases with high *CYB5R1* expression are significantly (t-test) more frequent among cases with lymph node metastasis (N+, C) and distant metastasis (M1, D), when compared to cases with low *CYB5R1* expression.

Since EMT is strongly implicated in carcinoma progression [12], we tested for clinical relevance of CYB5R1 expression in CRC. In our collection of 221 CRCs, CYB5R1 expression scores (Figure 1) strongly separated patients with good (score 0, five-year survival rate 97%), moderate (scores 1 and 2, five-year survival rates 80% and 74%, respectively), and poor (score 3, five-year survival rate 25%) cancer specific survival at an inter-observer agreement of κ=0.56 (Figure 5A). Testing for disease free survival yielded similar, yet slightly less stark results (Figure 5B). Based on these findings and due to low frequency of cases with CYB5R1 score 3, we then re-classified cases into CYB5R1 negative (score 0) and CYB5R1 positive (scores 1-3) categories only (inter-observer agreement κ=0.69). Again, Kaplan-Meier statistics revealed significantly worse cancer specific survival and marginally worse disease free survival of CYB5R1 positive cases (Figures 5C, 5D). Next, we evaluated co-occurrences of CYB5R1 expression with other clinical/pathological variables. CYB5R1 positivity was associated with low tumor grade and was more frequent in cancers of the left colon or rectum, whereas no correlations with age, gender, or T-category were found (Table 1). Including these variables into a proportional hazards regression analysis revealed that CYB5R1 positivity was an independent predictor of poor tumor specific survival in CRC, indicating a high relative risk (hazard ratio 8.5, Table 2). Finally, to independently validate these findings, we tested for clinical correlations of *CYB5R1* mRNA levels in the TCGA data set of 457 colon cancers. Using ROC curve analyses, we determined an optimal cutoff score of 584.5 normalized mRNA reads (Figure 6A), and dichotomal classification of cases by this score revealed a strong positive correlation of high *CYB5R1* expression and poor cancer specific survival in Kaplan-Meier statistics (Figure 6B). In line with these findings, cases with high *CYB5R1* expression also significantly correlated with lymph node metastasis (Figure 6C) and distant metastasis (Figure 6D). Again, proportional hazards regression analyses including key clinical variables proved independent prognostic power of *CYB5R1* expression in this data set (Table 3). Collectively, CYB5R1 expression is a strong and independent marker of poor prognosis in CRC.

**Table 1:** Clinicopathological variables and correlation with CYB5R1

Characteristics	Total	CYB5R1		p
		positive	negative	
All patients	221 (100)	190 (86.0)	31 (14.0)	
Age (y, Median 69)				
≤ 68	111 (50.2)	96 (86.5)	15 (13.5)	0.825
≥ 69	110 (49.8)	94 (85.5)	16 (14.5)	
Gender				
Male	123 (55.7)	110 (89.4)	13 (10.6)	0.097
Female	98 (44.3)	80 (81.6)	18 (18.4)	
Tumor location				
Right hemicolon	72 (32.6)	52 (72.2)	20 (27.8)	<0.01
Left hemicolon	103 (46.6)	98 (95.1)	5 (4.9)	
Rectum	38 (17.2)	32 (84.2)	6 (15.8)	
Unknown	8 (3.6)	8 (100.0)	0 (0.0)	
T stage (UICC)				
T1	1 (0.5)	1 (100)	0 (0.0)	0.469
T2	35 (15.8)	28 (80.0)	7 (20.0)	
T3	177 (80.1)	153 (86.4)	24 (13.6)	
T4	8 (3.6)	8 (100.0)	0 (0.0)	
Tumor grade (WHO)				
low grade	199 (90)	175 (92.1)	24 (77.4)	0.011
high grade	22 (10.0)	15 (7.5)	7 (22.6)	

Values in parentheses indicate column and row percentage for total and CYB5R1 positive or negative cases, respectively.

**Table 2:** Multivariate analysis of cancer specific survival, tissue collection

Variables	Cancer specific survival		p
	HR	(95% confidence interval)	
Age ≥ median (69 y)	1,85	(0.99-3,44)	0.053
Female vs. male	0,73	(0.363-1.461)	0.37
Rectal location	4,32	(2.06-9.04)	<0.01
T stage	3,93	(1.77-8.76)	<0.01
High tumor grade	1,37	(1.37-3.24)	0.47
CYB5R1 positive	8,51	(1.13-64.05)	0.038

**Table 3:** Multivariate analysis of cancer specific survival, TCGA collection

Variables	Cancer specific survival		p
	HR	(95 % confidence interval)	
Age ≥ median (68 y)	0.75	(0.35-1.62)	0.46
Female vs. male	1.30	(0.35-1.67)	0.51
T stage	5.05	(2.25-11.34)	<0.001
Nodal metastasis	4.13	(1.62-10.54)	0.003
MSI high	0.73	(0.21-2.51)	0.62
CYB5R1 high	2.29	(1.05-5.02)	0.038

## DISCUSSION

The ability to assume different morphologies and phenotypes within the same core genetic background implies substantial tumor cell plasticity of colorectal cancer cells [19]. In this context, EMT might be the most significant phenotypic switch, allowing tumor cells at the leading edge to lose cellular junctions and polarity, with acquisition of migratory and invasive properties that eventually enable tumor spread from original to metastatic sites [9, 20]. Characterizing CRC cells that undergo EMT therefore appears strategically important to identify drug-targetable weaknesses of this infiltrative tumor cell subset, envisioning the development of more effective therapeutic strategies, especially for late stage disease [15]. In addition, determining the degree of general EMT potential in CRCs may allow separating more and less aggressive tumors with subsequent adjustment of clinical management.

Here, we establish CYB5R1 as a potential marker for CRC cells of an infiltrative EMT like tumor cell phenotype which can be used to easily visualize this tumor cell subset *in situ* by immunostaining in primary colon cancer tissues. Markers that indicate EMT in tissues are sparse, and detection of typical EMT markers such as ZEB1, SNAIL1, or Vimentin is difficult in primary colon cancers, as reflected by few convincing *in situ* studies [21, 22]. Additionally, interpretation of EMT marker expression often is confounded by strong labelling of tumor-surrounding stromal cells in which tumor cells undergoing EMT may be difficult to identify [23]. CYB5R1 apparently overcomes some of these restrictions by most strongly labelling tumor cells at the leading tumor edge, with little or absent expression in surrounding stromal cells. Nevertheless, CYB5R1 staining is not absolutely exclusive to infiltrative dissociating tumor cells but extends to the glandular tumor cell compartment, especially in cases with strong CYB5R1 expression, which mirrors limited exclusiveness of other core EMT markers for morphologically dissociating CRC cells, such as ZEB1 and SNAIL1 [21, 23]. Therefore, it remains to be determined to what extent infiltrative tumor cell morphology and EMT phenotypes indicate identical tumor cell subpopulations in CRC.

The link between CYB5R1 and EMT is greatly strengthened by our data from gene set enrichment analyses (GSEA), which revealed an extraordinarily strong correlation of *CYB5R1* mRNA expression, and the expression of two core EMT gene signatures [17, 18] in a large independent data set from the TCGA. This not only implies that CYB5R1 may be specifically useful to determine the overall degree of EMT and cellular plasticity of individual colon cancers, regardless of its intratumoral distribution. It may also indicate significant CYB5R1 enzymatic activity within colon cancer cells undergoing EMT. *CYB5R1* is part of a family of genes encoding for redox enzymes that are involved in the transfer of reducing equivalents from nicotinamide adenine dinucleotide (NADH). These enzymes are found in soluble and in membrane bound forms, and the latter are expressed in various tissues where they are involved in oxidative stress protection, prevention of apoptosis and detoxification processes [24, 25], but also in drug metabolism of carcinogenic and anticancer drugs [16, 26]. While this suggests that colon cancer cells with high CYB5R1 expression undergoing EMT are highly metabolically active, specifically resistant to apoptosis, and protected from carcinogenic mutagens, our findings have important implications for a potential targetability of this tumor cell subset. The activation of amidoxime prodrugs to amidines requires mitochondrial activity of cytochrome b5, its reductases, and mitochondrial amidoxime reducing component (mARC) [27]. If CYB5R1 expression indicates high activity of this metabolic pathway, we hypothesize that this may open opportunities for specifically designed amidoxime drugs targeting EMT in CRC. Moreover, since our *in vitro* data suggested dependence of migratory and invasive colon cancer cell phenotypes on CYB5R1 expression, inhibition of this enzyme may as well hold potential for therapeutic intervention. In such contexts CYB5R1 might be an easily assessable biomarker on the tissue level, in addition to indicating EMT. However, therapeutic interference with cytochrome reductases in cancer will require careful evaluation, since for certain entities tumor suppressor functions of these enzymes were recently reported [28].

EMT is considered regulator of invasion and metastasis and thus cancer progression [12], and our data on CYB5R1 provide additional evidence for this concept. When looking at clinical correlates in our collection of 221 early stage CRCs with prospectively recorded follow-up data, stratified for survival assessment, CYB5R1 proved to be a useful predictor of poor prognosis. However, since prognostic markers tested in individual case collections can be of limited value, we subsequently validated our findings in the large data set of colon cancers from the TCGA, where high *CYB5R1* expression also was linked to earlier tumor dependent death, as well as to nodal and distant metastasis. Importantly, in both case collections, poor survival prediction by CYB5R1 protein or mRNA was independent of other key clinical variables, and thus could be useful to complement cancer staging, when evaluating patient prognosis [29]. High CYB5R1 expression may therefore identify CRC patients in need of increased clinical attention that may benefit from more aggressive or adjuvant treatment strategies. We suggest that this independent prognostic power of CYB5R1 is due to gauging EMT, not significantly reflected in other clinical variables. Nevertheless, before clinical implementation of CYB5R1 as a prognostic marker, our findings will require further independent validation. Collectively, we here demonstrate an important link of CYB5R1, EMT and colon cancer progression that might be exploited for diagnostic and eventually therapeutic purposes in CRC patients.

## MATERIALS AND METHODS

### Clinical samples

CRC specimens from patients that underwent intentionally curative surgical resection between 1994 and 2006 at the LMU were drawn from the archives of the institute of pathology. Follow-up data were recorded by the Munich Cancer Registry. Specimens and data were anonymized, and the need for consent was waived by the institutional ethics committee of the Medical Faculty of the Ludwig-Maximilians-Universität München (LMU). Inclusion criteria were patients with localized colorectal adenocarcinomas and absence of nodal (N0) or distant metastasis (M0) at the time of diagnosis (UICC stage I and II [30]). None of the patients received adjuvant therapies. Staging and grading was reviewed for all cases. Tumor tissues were assembled into tissue microarrays (TMAs) with representative 1 mm cores, including triplicates of tumor edges and tumor centers of each case. The final collection consisted of 221 CRC cases of which in 43 cases (19%) patients had died of their tumor within the follow-up period. For tumor specific survival analysis, CRC attributed deaths were defined as clinical endpoints. For analysis of disease free survival, tumor progression after surgical resection was the clinical endpoint, documented as either tumor recurrence or metastasis. Cancer specific survival was analyzed by the Kaplan-Meier method and groups were compared with the log-rank test. Cox proportional hazards model was used for multivariate analysis. Statistics were calculated using SPSS (IBM).

### Immunohistochemistry, immune fluorescence and confocal microscopy

For immunohistochemistry, 5 μm TMA sections were cut, deparaffinized, and stained with rabbit anti-CYB5R1 (Sigma, HPA010641, 1:200) polyclonal antibody (Ab) on a Ventana Benchmark XT autostainer with ultraView Universal DAB detection kits (Ventana Medical Systems). Staining was evaluated independently by two observers that were blinded from clinical outcome. For each case, CYB5R1 staining was categorized into complete absence of staining (negative, score 0), low intensity staining of tumor cells (weak expression, score 1), high intensity staining of less than 50 % of tumor cells (moderate expression, score 2), and high intensity staining of 50 % or more tumor cells (strong expression, score 3). Inter-observer agreement was calculated by κ-statistics [31]. For immune fluorescence, 5 μm whole tissue sections were deparaffinized and antigens were retrieved in TRS6 (Dako Cytomation) for 20 min in a microwave oven. Slides then were incubated sequentially with anti-CYB5R1 Ab and mouse anti-Laminin gamma 2 monoclonal Ab (Milipore, D4B5, 1:200), or mouse anti-E-Cadherin monoclonal Ab (Santa Cruz, 1:50) for 1 h each at room temperature, washed with PBS, and then with AlexaFluor 565 conjugated donkey anti-rabbit (Abcam, 1:500) and AlexaFluor 488 conjugated goat anti-mouse (Invitrogen; 1:500). Nuclei were counterstained with DAPI (Vector Laboratories). Confocal fluorescence images were taken on a LSM 700 laser scanning microscope using the ZEN software (Carl Zeiss). Isotype-controls were included for all antibodies.

### TCGA colon cancer data

Gene expression (RNA-Seq) data and corresponding clinical data of 457 colon cancer samples were retrieved from The Cancer Genome Atlas (TCGA) database (https://tcga-data.nci.nih.gov/tcga/). Patients that died with tumor being present were defined as the clinical endpoint for tumor specific survival. For binary classification of cases, receiver operated characteristics (ROC) curve analysis were used to determine optimal *CYB5R1* cutoff values. Kaplan-Meier curves, statistics and multivariate analyses were done as described above.

### Gene set enrichment analyses (GSEA) and heat maps

Pearson correlations of *CYB5R1* expression and expression of 20,531 genes within the TCGA data set were calculated and genes were ranked accordingly. GSEA analyses were conducted using this ranked gene list against C2 curated gene sets from the Molecular Signatures Database v5.0 (Broad Institute). The default parameters of GSEA with gene lists of 15 to 500 genes were used and analyses were run with 1,000 permutations. Heat maps and clustering for individual EMT factors were calculated with the GENE-E software (Broad Institute).

### Cell culture, immunoblotting, migration and invasion assays

DLD-1 and HCT116 colon cancer cell lines were obtained from the American Type Culture Collection and maintained in DMEM containing 10 % FBS, 100 U/ml penicillin, and 0.1 mg/ml streptomycin (Biochrom). For transient CYB5R1 knockdown or control transfection, pre-designed siRNAs were purchased from Thermo Fisher Scientific, and transfected into both cell lines at 10 nM final concentration using HiPerFect (Qiagen).

For immunoblotting, transfected colon cancer cells were harvested after 48 h, whole cell lysates were supplemented with protease and phosphatase inhibitors (Roche), and blotted onto PVDF membranes (Merck Millipore). Membranes then were incubated with rabbit anti-CYB5R1 polyclonal Ab (1:1000) or mouse anti-Tubulin monoclonal Ab (1:50000, Sigma). Bands were visualized using HRP-conjugated secondary mouse (Promega) or rabbit (Sigma) Ab and Chemiluminescent HRP Substrate (Millipore).

For transwell migration and invasion assays, 8 μm ThinCert cell culture inserts (Greiner Bio-One) were used, which for invasion were coated with 100 μl of 1 mg/ml growth factor depleted Matrigel (Corning). 1×10^5^ siRNA transfected cells per well were seeded in serum free medium in the upper chamber of the inserts, 500 μl serum-free medium was added to the lower chamber, and replaced 24 hours later by DMEM with 10 % FBS. Inserts were removed after 1 day for migration and 3 days for invasion. Cells were fixed in 4 % paraformaldehyde and methanol, and stained with crystal violet. Residual cells from the upper chamber were removed with cotton swabs, and photomicrographs of migrated or invaded cells were taken. For quantification, staining from culture inserts was dissolved in 250 μl of 30 % acetic acid, and absorbance was measured at 590 nm on a Varioskan instrument (Thermo Scientific).
